# 284 Effect of <20-minutes of Cool Running Water < 3hours on Burn Patient Outcomes: A Systematic Review

**DOI:** 10.1093/jbcr/irad045.259

**Published:** 2023-08-29

**Authors:** Yvonne M Singer

**Affiliations:** Victorian Adult Burn Service, Melbourne, Victoria

## Abstract

**Introduction:**

Burn injuries are a leading cause of morbidity that can result in devastating disability and poor quality of life for survivors. This systematic review aimed to synthesise evidence regarding the effect of 20 minutes of cool running water (CRW) within three hours of injury on outcomes of patients with thermal burn injuries.

**Methods:**

This systematic review was conducted in reference to the Preferred Reporting Items for Systematic Reviews and Meta-Analyses. Multiple databases (PubMed, EMBASE, CENTRAL, CINAHL Complete via EBSCO, PROQUEST Dissertations and Theses), and the Australia New Zealand Clinical Trial Registry were searched for eligible studies published in English and Chinese, without date restriction. Meta-analyses were undertaken. Methodological quality of studies was assessed by using Downs and Black Checklist.

**Results:**

Of 323 records, seven studies were included, with a combined study population of 11,383 patients. The majority (67%) of studies were conducted in Australia and New Zealand. The methodological quality was ranked between ‘fair’ and ‘good’. Approximately half of the study population (n=5782 [50.8%]) received 20 minutes of CRW within three hours of injury. Patients who received 20 minutes of CRW, within three hours of injury, had significantly decreased odds of requiring skin grafting or other surgical intervention when compared to patients who did not receive 20 minutes of CRW within three hours of injury.

**Conclusions:**

There is considerable evidence demonstrating the association between the application of 20 minutes of CRW within the first three hours of injury and improved outcomes for patients with burn injury. Burns first aid guidelines and recommendations vary widely globally. Burn care organisations should reach international consensus about burns first aid best practice and provide clear, simple, and unified messaging. Burn care organisations should also engage in cross-sector collaborations with relevant jurisdictional EMS organisations to translate burns first aid evidence into pre-hospital practice.

**Applicability of Research to Practice:**

Applying CRW after burn injury is a relatively simple procedure that can be performed at the scene by conscious patients, bystanders, firefighters, and other EMS responders. Data from the ABA burn repository shows that burn injuries in the United States (US) mostly occur in the home and most are < 10% total body surface area (TBSA). Therefore, for US EMS responders responding to the burn injured, CRW is accessible in most situations, when injuries occur in the home and the medical urgency to transfer most patients with non-severe injuries (< 10%TBSA) is not time critical. These contextual conditions of responding to most people with burn injuries, uniquely places US EMS responders in a window of opportunity to provide CRW at the scene, that can significantly reduce the burden of injury for patients.

